# Double-wire technique to facilitate vein of Marshall cannulation and ethanol infusion in atrial fibrillation: a case series

**DOI:** 10.1186/s12872-023-03553-9

**Published:** 2023-10-24

**Authors:** Hong-Da Zhang, Lei Ding, Kuo Zhang, Feng-Yuan Yu, Li-Jie Mi, Si-Xian Weng, Zi-Han Jiang, Min Tang

**Affiliations:** 1 Arrhythmia Center, State Key Laboratory of Cardiovascular Disease, Fuwai Hospital, National Center for Cardiovascular Diseases, Chinese Academy of Medical Sciences & Peking Union Medical College, 167 Beilishi Road, Xicheng District, Beijing, 100037 China; 2 National Center for Clinical Laboratories, Institute of Geriatric Medicine, Beijing Hospital, Chinese Academy of Medical Sciences, National Center of Gerontology, Beijing, China

**Keywords:** Atrial fibrillation, The vein of Marshall, Ethanol infusion, Cannulation, Case report

## Abstract

**Background:**

The vein of Marshall (VOM) ethanol infusion is increasingly performed in combination with catheter ablation in atrial fibrillation (AF). The cannulation of the VOM can sometimes be challenging. This study aimed to evaluate the double-wire technique in cases of difficult cannulation of the VOM.

**Case presentation:**

Patients with AF scheduled for combined catheter ablation and VOM ethanol infusion were consecutively enrolled. The procedure was performed via the femoral vein. If the regular cannulation technique with one angioplasty wire failed or took more than 20 min, the double-wire technique using a stabilizing wire and a cannulation wire was performed. The unique technique was used mainly in two scenarios, when the Eustachian ridge was too prominent as a barrier for catheter manipulation or when the VOM ostium was close to the coronary sinus ostium. Of 162 patients scheduled for VOM ethanol infusion, the double-wire technique was applied in 6 (3.7%) patients and led to a 100% successful cannulation rate of the VOM. Of the six patients, two had a prominent Eustachian ridge, and four had a VOM ostium close to the coronary sinus ostium. The mean cannulation time was 33.3 ± 7.3 min. The ethanol infusion was successfully performed in 5 patients. One patient had a collateral circulation in the distal VOM, and ethanol infusion was not performed.

**Conclusions:**

The double-wire technique can facilitate VOM cannulation and ethanol infusion in challenging cases.

**Word count:**

: 231.

## Introduction

The vein of Marshall (VOM) is not simply a remnant of the left superior vena cava. Its surrounding area contains sympathetic nerve fibers, ganglia, blood vessels, and multiple myocardial tracts [1, 2]. Studies show that VOM plays a vital role in atrial fibrillation (AF), and VOM ethanol infusion improves sinus rhythm maintenance in patients with AF. [2–7].

Unlike catheter ablation of AF, VOM ethanol infusion requires more knowledge of the fluoroscopic anatomy and angioplasty tool. The success rate of VOM ethanol infusion varies in different studies from 83 to 93%. [5, 7] The VOM ethanol infusion procedure consists of two key steps, the identification, and cannulation of the VOM. There have been many methods reported to increase the identification rate of VOM. [7–10] However, the cannulation technique is scarcely reported. In our experience, there are some scenarios in which the VOM is difficult to cannulate. The present study aimed to introduce a unique double-wire technique to facilitate VOM cannulation in these problematic cases.

## Case Presentation

**Fig. 1:**
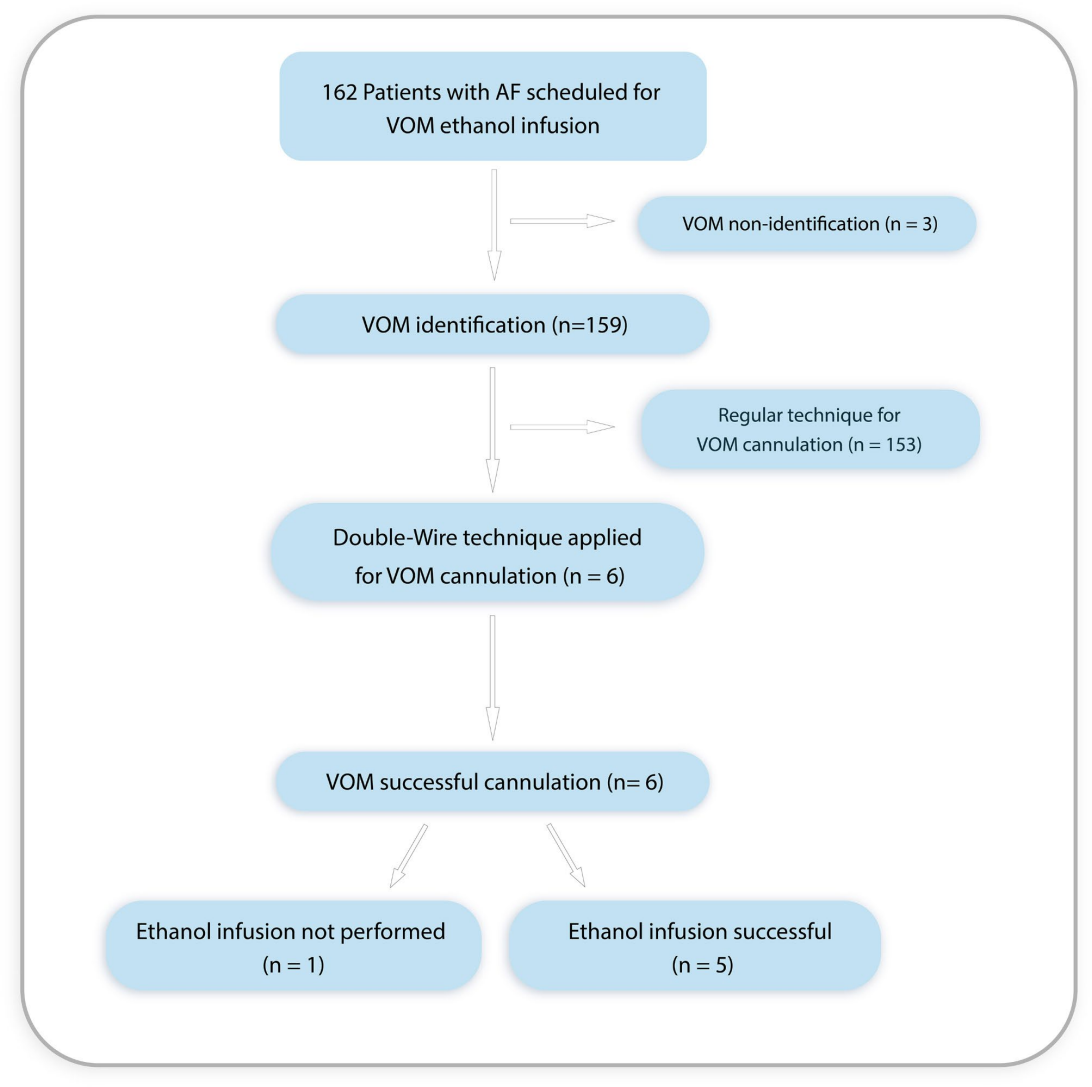
Flow chart of patient enrollment. AF, atrial fibrillation; VOM, the vein of Marshall

Patients with AF who underwent radiofrequency catheter ablation and VOM ethanol infusion in Fuwai Hospital, Beijing, China, between November 2021 and September 2022 were consecutively enrolled. Patients with persistent AF who underwent the double-wire technique for VOM ethanol infusion were included in this study (Fig. 1). Regular VOM ethanol infusion only needs one angioplasty wire for VOM cannulation. The double-wire technique used two wires during the procedure, one for stabilizing the guiding catheter position (the stabilizing wire) and the other for VOM cannulation (the cannulation wire). This study was performed in accordance with the Declaration of Helsinki and was approved by the Review Board and Ethics Committee of Fuwai Hospital (Approval No. 2022 − 1810). Informed consent was obtained from all participants.

The overall procedure included the following steps: (1) pulmonary vein isolation; (2) coronary vein angiography for VOM identification; (3) determination of strategy of VOM cannulation; (4) VOM cannulation and ethanol infusion; (5) catheter ablation of the mitral isthmus. The technical details of catheter ablation have been previously reported. [9] After catheter ablation, a guiding catheter (6-F Judkins Right [JR] 4; Medtronic, Minneapolis, MN, USA) was positioned inside the coronary sinus (CS) through an SL1 long sheath (8.5 F; St. Jude Medical, Inc., St Paul, MN, USA) or a steerable long sheath (8.5-F, Agilis NxT; Abbott, St Paul, MN, USA) inserted from the right femoral vein. *The coronary vein angiography was performed in the right anterior oblique (RAO) 30°, the left anterior oblique (LAO) 30°, and the LAO cranial views (30°) for VOM identification* [9].

If the VOM is identifiable, the cannulation strategy was discussed and determined by two experienced operators. The double-wire technique is a commonly used technique in coronary artery interventions. *We believe it is reasonable and safe to be used in coronary vein interventions.* In this study, this technique was used when the regular cannulation technique with one angioplasty wire failed or took more than 20 min. This usually happened in two scenarios, when the Eustachian ridge was too prominent as a barrier for catheter manipulation or when the VOM ostium was close to the CS ostium. For the double-wire technique, the first angioplasty wire (the stabilizing wire, Sion Blue 0.014 inch, Asahi) was first positioned as distal as it could in a distal branch of the CS, which was the anterior interventricular vein in most cases. Then an over-the-wire angioplasty balloon (1.5–2.5 mm diameter and 8–12 mm length; Boston Scientific, Cambridge, MA, USA) preloaded with a second angioplasty wire (the cannulation wire, Sion Blue 0.014 inch, Asahi) was advanced into the VOM through the guiding catheter. After successful VOM cannulation, the balloon was inflated, and ethanol was delivered at 2–3 positions distally to proximally in the VOM. The cannulation time was defined as the time from the beginning of coronary vein angiography to successful VOM cannulation. After ethanol infusion, catheter ablation of the mitral isthmus was performed. All the procedures were performed by Dr. Min Tang.

A flow chart of patient enrollment is shown in Fig. 1. A total of 162 patients were enrolled, 159 of whom with an identifiable VOM had an ethanol infusion attempt (Fig. 1). Six patients underwent the double-wire technique for VOM cannulation, and all succeeded. Further ethanol infusion was successfully performed in 5 patients. One patient had a collateral circulation in the distal VOM, and ethanol infusion was not performed. The baseline characteristics of these six patients are displayed in Table 1. They all had persistent AF. The mean cannulation time was 33.3 ± 7.3 min. Patients had a mean age of 52 ± 14 years old, and half were female. The mean left atria dimension and left atria volume were 44 ± 3.5 mm and 80 ± 21 ml, respectively.

**Table 1:** Patient characteristics and procedural data

Patient No.	Age, y	Sex	BMI, kg/m^2^	Comorbidities	CHA_2_DS_2_-VASc score	HAS-BLED score	LA, mm	LAV, ml	LVEF, %	Cannulation time, min	Ethanol infusion success
1	57	Female	27.78	HTN	2	0	41	63	63	26	Yes
2	67	Female	29.30	CAD	2	1	42	72	58	37	No
3	69	Male	20.83	HF	2	1	48	90	58	23	Yes
4	52	Male	26.83		0	0	49	97	61	39	Yes
5	62	Male	33.46	HTN, DM, HF	4	1	41	52	58	41	Yes
6	60	Female	31.51	HTN, Stroke	4	2	44	106	60	34	Yes

BMI, body mass index; HTN, hypertension; CAD, coronary artery disease; HF, heart failure; DM, diabetes mellitus; CHA_2_DS_2_-VASc: congestive heart failure, hypertension, age ≥ 75 years, diabetes mellitus, stroke, vascular disease, age 65–74 years, sex category (female); HAS-BLED: hypertension, abnormal renal/liver function, stroke, bleeding history or predisposition, labile international normalized ratio, elderly (> 65 years of age), concomitant drugs/alcohol; LA, left atria,; LAV, left atrial volume; LVEF, left ventricular ejection fraction

**Fig. 2:**
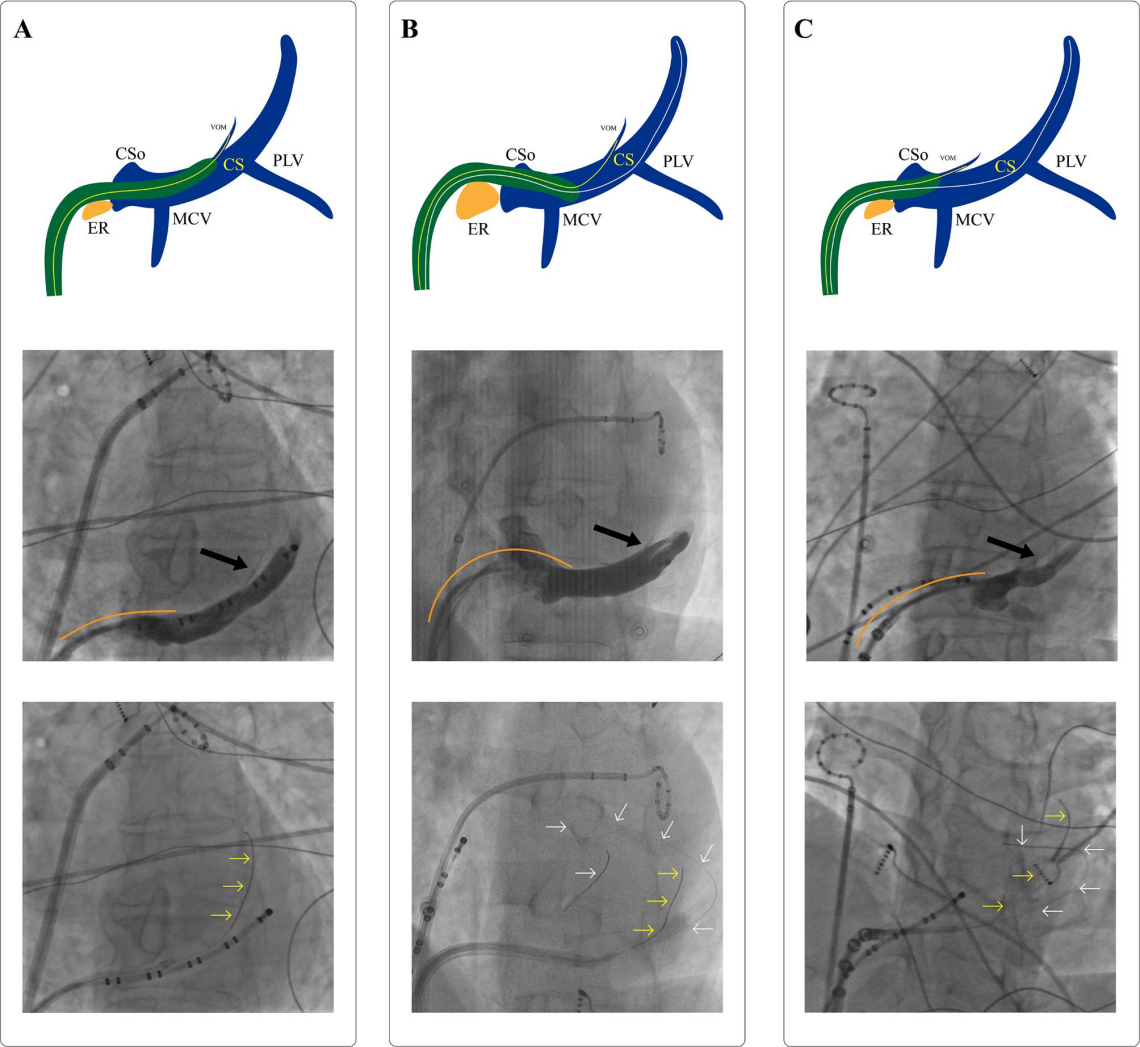
Different scenarios suitable for the regular and double-wire technique. **A**, the scenario that does not need the double-wire technique; **B**, the scenario suitable for the double-wire technique because the Eustachian ridge was too prominent as a barrier for catheter manipulation; **C**, the scenario suitable for the double-wire technique because the VOM ostium was close to the CS ostium. The figures are all shown in the left anterior oblique view. VOM, the vein of Marshall; CS, coronary sinus; CSo, CS ostium; MCV, middle cardiac vein; PLV, posterior lateral vein; ER, the Eustachian ridge. The black arrow indicates the VOM; the yellow arrow indicates the cannulation wire in the VOM; the white arrow indicated the stabilizing wire in the CS; the orange curve indicates the shape of the guiding catheter above the ER

**Fig. 3:**
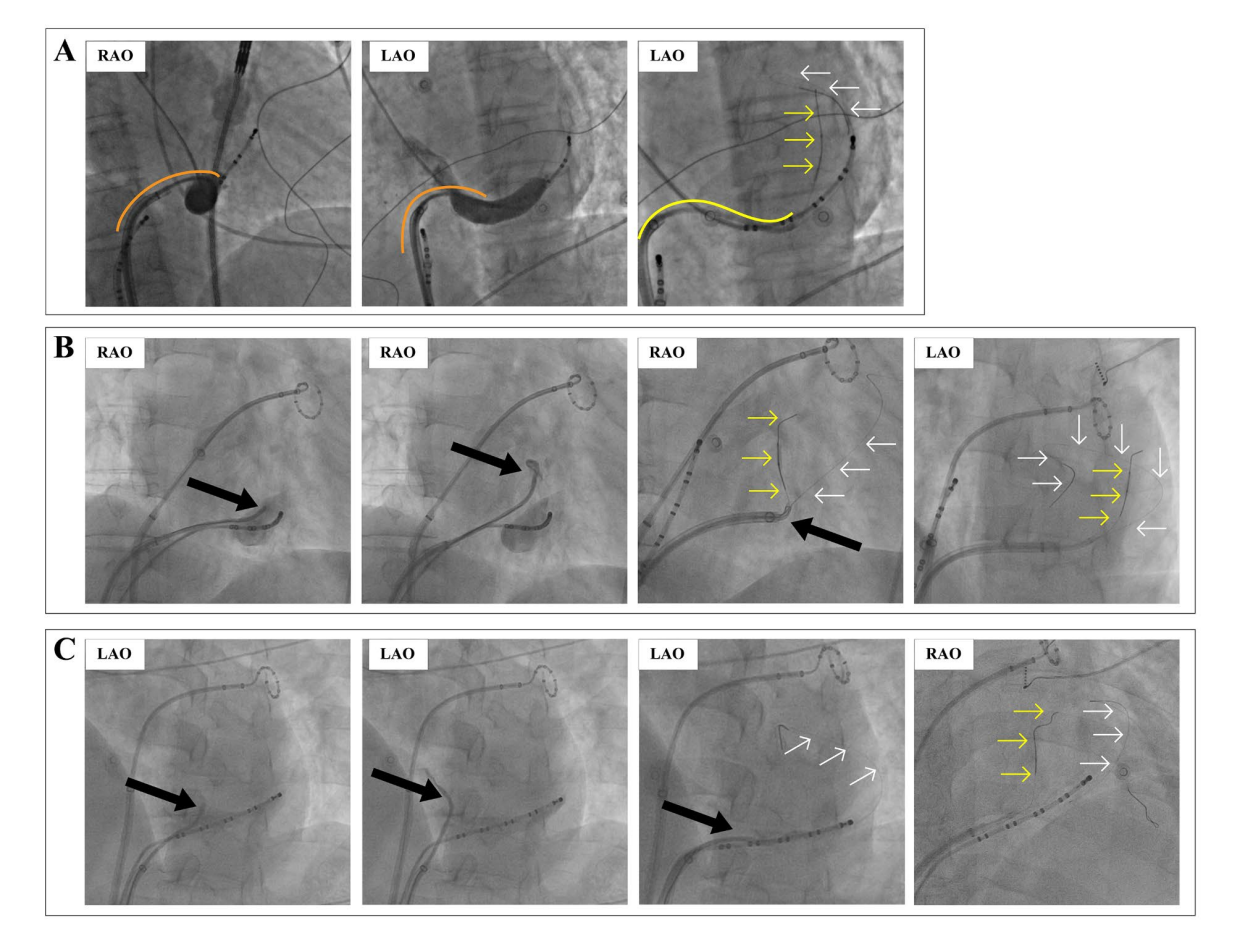
Examples of the double-wire technique. **A**, the double-wire technique used in a patient with prominent Eustachian ridge. **B** and **C**, the double-wire technique used in patients with VOM ostium close to the CS ostium. RAO, right anterior oblique; LAO, left anterior oblique; VOM, the vein of Marshall; CS, coronary sinus. The black arrow indicates the VOM; the yellow arrow indicates the cannulation wire in the VOM; the white arrow indicated the stabilizing wire in the distal CS; the orange curve indicates the shape of the guiding catheter above the ER; the yellow curve indicated a reversed S-shape of the guiding catheter

Figure 2B-C shows the two scenarios in which the double-wire technique can be applied compared with the regular single-wire technique used in most patients, as shown in Fig. 2A. In this study, two patients had a prominent Eustachian ridge (Fig. 2B), and four had a VOM ostium close to the coronary sinus ostium (Fig. 2C). Figure 3 A shows that in a patient with a prominent Eustachian ridge, the guiding catheter was in a reverse-S shape which was unstable. The stabilizing wire helped stabilize the whole system for successful cannulation. In two patients with VOM ostium close to the CS ostium, the guiding catheter slipped out of the CS lumen at the initial attempt and then was stabilized with the help of the stabilizing wire (Fig. 3B-C).

**Fig. 4:**
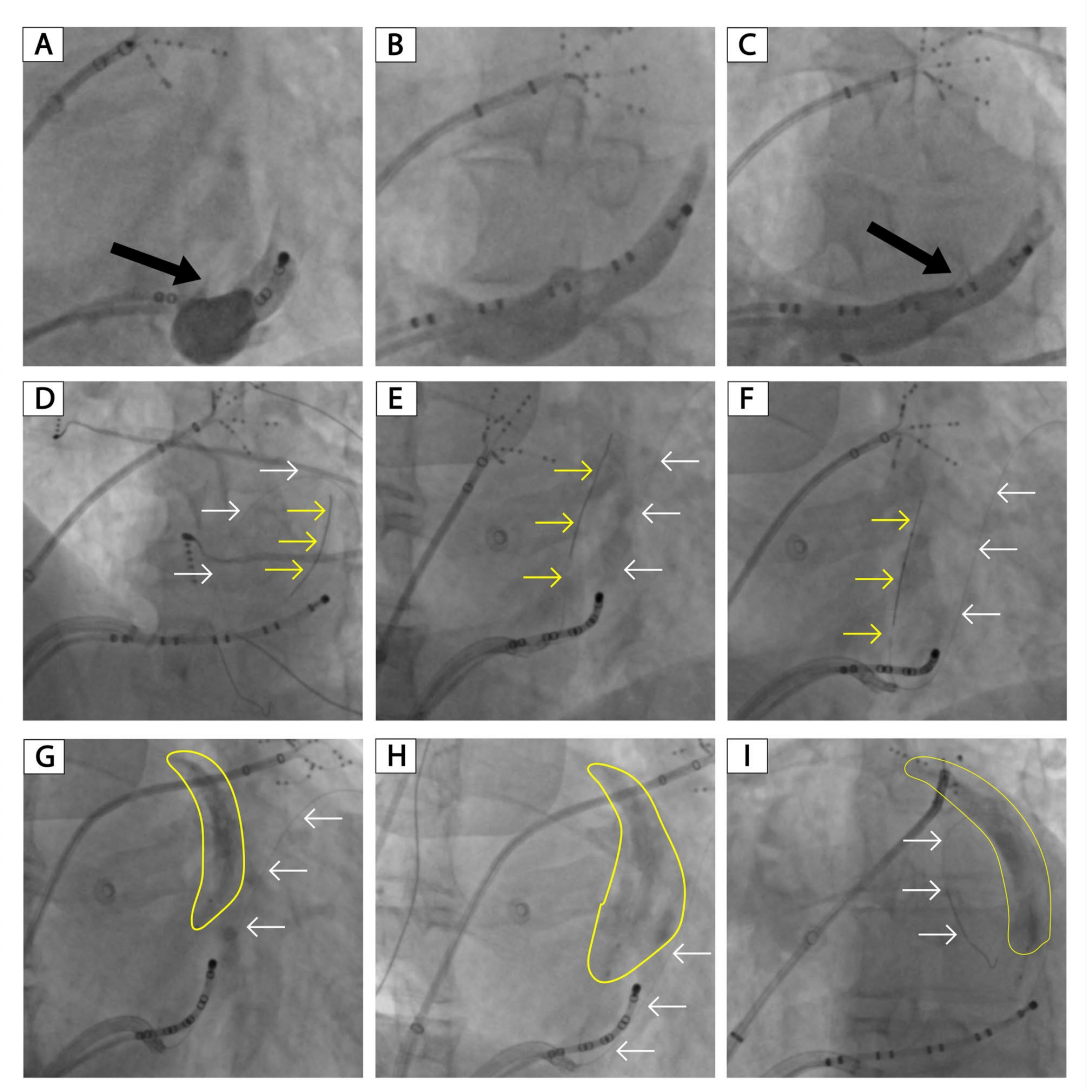
Procedural process for the double-wire technique. **A-C**, Coronary vein angiography in the RAO, LAO, and LAO cranial view for VOM identification. D-E, the first wire (the stabilizing wire) and the second wire (the cannulation wire) were seen in the LAO cranial view and verified in the RAO view. F, the angioplasty balloon was advanced in the VOM. G-I, localized staining in the RAO, LAO, and LAO cranial view. The black arrow indicates the VOM; the yellow arrow indicates the cannulation wire in the VOM; the white arrow indicated the stabilizing wire in the distal CS; the yellow circle indicates the localized staining

Figure 4 shows the procedure process step by step. The CS angiography was performed in the RAO, LAO, and LAO cranial view to identify the VOM (Fig. 4A-C). In this patient, the VOM could be identified in the RAO and LAO cranial view but not in the LAO view. The first (the stabilizing wire) was positioned as distal as possible in the CS, probably in the distal anterior interventricular vein. The second wire (the cannulation wire) was advanced into the VOM in the LAO cranial view and verified in the RAO view (Fig. 4D-E). Then the angioplasty balloon was advanced in the VOM (Fig. 4F). Finally, 8 ml ethanol was infused in the VOM, and the localized staining could be seen in all three views (Fig. 4G-I).

**Fig. 5:**
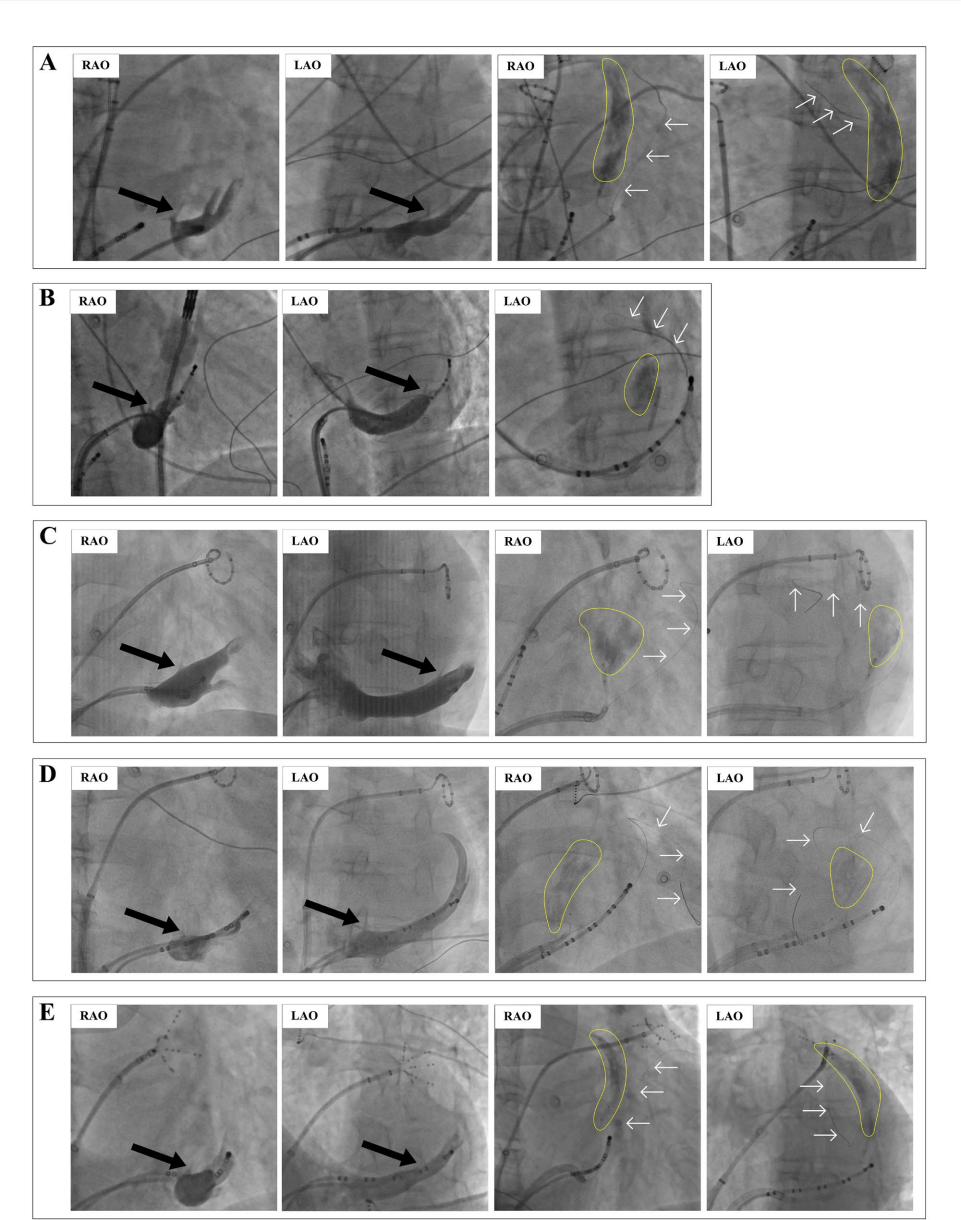
The angiography of patients with successful ethanol infusion. **A-E**, angiography of the five patients (Patients No. 1, 3, 4, 5, and 6) with successful ethanol infusion. RAO, right anterior oblique; LAO, left anterior oblique. The black arrow indicates the VOM; the white arrow indicated the stabilizing wire in distal CS; the yellow circle indicates the localized staining

In 5 patients, VOM ethanol infusion was successful with manifest localized staining (Fig. 5). The VOM ostium was proximal to the CS ostium in 3 patients, as shown in Fig. 5A, D-E. In this study, the distance from the VOM ostium to the CS ostium in the LAO and LAO cranial views, and the diameter of the VOM ostium and CS ostium in all three views were recorded (Table 2). The distance from the VOM ostium to the CS ostium in the LAO cranial view in the three patients (Patients No. 1, 5, and 6) was 11.3, 12.4, and 13.7 mm, respectively (Table 2). The distance for the other two patients (Patients No. 3 and 4) was 27.2 and 43 mm, respectively. These two patients had a prominent Eustachian ridge, as shown in Fig. 5B-C.

**Table 2:** Angiography results

**Patient No.**	**RAO view**		**LAO view**			LAO cranial view		
	VOMo diameter, mm	CSo diameter, mm	VOMo diameter, mm	CSo diameter, mm	Distance from VOMo to Cso, mm	VOMo diameter, mm	CSo diameter, mm	Distance from VOMo to Cso, mm
1	2.3	10.9	2.0	10.9	8.8	2.2	11.2	11.3
2	1.6	13.0	1.7	11.8	24.3	1.7	13.4	25.0
3	1.6	10.3	1.6	10.0	25.2	1.7	11.4	27.2
4	2.5	16.6	2.1	13.6	42	2.2	15.4	43.0
5	1.5	14.5				1.6	14.8	12.4
6	2.0	13.5				1.9	15.1	13.7

RAO, right anterior oblique; LAO, left anterior oblique; VOM, the vein of Marshall; VOMo, VOM ostium; CS, coronary sinus; CSo, CS ostium

## Discussion

This study introduces a new method of VOM cannulation in challenging cases. The results showed that the double-wire technique was feasible with a high cannulation success rate in patients with a prominent Eustachian ridge or a VOM ostium close to the CS ostium.

There are variabilities in the characteristics of the VOM, including the distance to the CS ostium, the width and length, branching patterns, and collateral circulation. [7, 9, 11] We conclude in our clinical practice that if the VOM is located too proximal to the CS ostium (Fig. 2C), the cannulation takes more time and could even fail. In this scenario, the sheath and guiding catheter could only be positioned on the edge of the CS ostium, which significantly decreases their stability. Sometimes the guiding catheters might slip out of the CS lumen (Fig. 3B-C), even with the help of a steerable sheath. And the ostium of the guiding catheter will be difficult to be rotated backward towards the VOM ostium. Using an additional angioplasty wire (the stabilizing wire) positioned distally in the distal CS can significantly improve the stability of the guiding catheter (Fig. 3C). Three patients in this study (Patients No. 1, 5, and 6) with a short distance from VOM to CS ostium underwent successful cannulation and ethanol infusion using the double-wire technique (Table 1; Fig. 5A, D-E).

The Eustachian ridge can be an obstacle to VOM ethanol infusion. A prominent Eustachian ridge may interfere with manipulating the sheath and guiding catheter (Figs. 2B and 3A). The guiding catheter would be in a reversed S-shape for VOM cannulation, which is unstable. A steerable sheath would help, but rotating the guiding catheter backward and upwards towards the VOM ostium is still difficult. An additional wire (the stabilizing wire) increases the stability of the guiding catheter during cannulation and ethanol infusion of the VOM. Two patients (Patients No. 3 and 4) in this study used the double-wire technique for this reason (Fig. 5B-C).

One patient (Patient No.2) in our study underwent successful cannulation of the VOM using the double-wire technique. But the venogram through the over-the-wire balloon showed distal collateral circulation. We decided not to perform further ethanol infusion during the procedure. Because the procedure was performed in our earlier stage of the project. We recently tried ethanol infusion in VOM with distal collateral circulation and succeeded. If we inject the ethanol as slowly as possible, the distal VOM, which is always the narrowest part, would be destroyed, and the distal collateral circulation would disappear. Then we can inject more ethanol, which could manifest localized staining in the VOM area.

Most patients in our clinical practice do not have a prominent Eustachian ridge, and the VOM location of whom is in the mid-to-distal CS, as shown in Fig. 2A. In this case, a regular technique with only one wire is enough. A regular technique might also succeed in complex cases, as shown in Fig. 2B-C. However, the double-wire technique introduced in this study provides an alternative that might shorten procedure time.

## Limitations

This retrospective analysis was conducted in a single center with only 6 cases using the double-wire technique. We perform VOM ethanol infusion with a high success rate in most cases using the regular technique. Some of the cases in this study might be solved via the internal jugular vein access, but it might increase the complexity of the procedure. Multicenter prospective studies are needed in the future.

## Conclusions

This study provides an alternative to the regular cannulation technique of the VOM. The double-wire technique using a stabilizing wire and a cannulation wire can facilitate the cannulation of VOM in challenging cases.

## Data Availability

Research data is confidential. Data sharing requests are required to meet the policies of the hospital and the funder. Please contact Dr. Min TANG (Email: doctortangmin@yeah.net) for Research data.
